# Transvaginal natural orifice transluminal endoscopic surgery for tubal ectopic pregnancy(vNOTESTEP): a protocol for a randomized controlled trial

**DOI:** 10.1186/s12884-025-07595-z

**Published:** 2025-04-23

**Authors:** Xinyu Xiao, Tianjiao Liu, Xin Li, Li He, Yonghong Lin, Dan Feng

**Affiliations:** 1 Department of Gynecology of Chengdu Women’s and Children’s Central Hospital, School of Medicine, University of Electronic Science and Technology of China, No. 1617, Riyue Avenue, Chengdu, Sichuan 610091 People’s Republic of China; 2 Department of Gynecology and Obstetrics, West China Second University Hospital, Sichuan University, Chengdu, China

**Keywords:** Natural orifice transluminal endoscopic surgery, Surgical complications, Minimally invasive surgery, Long-term postoperative outcomes, Female sexual function index, Childbirth

## Abstract

**Background:**

Tubal ectopic pregnancy is a life-threatening condition in early pregnancy. Minimally invasive laparoscopic surgery is increasingly used for the treatment of this disease. Retrospective studies suggest that Transvaginal Natural Orifice Transluminal Endoscopic Surgery (vNOTES) offers lower perioperative complications, faster recovery, and better cosmetic outcomes compared to other approaches. However, the lack of comprehensive perioperative and long-term postoperative data limits its widespread adoption in gynecology.

**Methods:**

The vNOTESTEP study is a randomized controlled trial (RCT) enrolling 72 patients requiring laparoendoscopic surgery for tubal ectopic pregnancy. After obtaining informed consent, preoperative assessments will be conducted. Following randomization, salpingectomy will be performed using either vNOTES or TU-LESS approach. The postoperative assessment and a structured 5-year follow-up, including eight visits, will be conducted. Baseline sociodemographic and clinical data will be collected from the Hospital Information System and patient interviews. Outcomes will be assessed perioperatively and postoperatively at designated time points (1st, 2nd, and 3rd postoperative day; 1st, 3rd, and 6th month; 1st–5th year). Key long-term outcomes include sexual function, pregnancy, vaginal delivery, and incisional hernia.

**Discussion:**

This RCT aims to provide robust clinical evidence on the perioperative and long-term outcomes of vNOTES versus TU-LESS for tubal ectopic pregnancy, focusing on key reproductive and surgical outcomes. The study seeks to refine patient selection criteria and contribute to guideline development for gynecologic vNOTES.

**Trial registration number:**

ChiCTR2400082909 (registered on April 10th, 2024).

**Supplementary Information:**

The online version contains supplementary material available at 10.1186/s12884-025-07595-z.

## Background

Ectopic pregnancy occurs when an embryo implants outside the uterine cavity, most commonly in the fallopian tubes [1]. It accounts for approximately 3% of all pregnancies and 10% of maternal morbidity, posing a significant threat to maternal health. Surgical intervention remains the primary treatment, with laparoscopy being the preferred approach due to its minimally invasive nature [2]. 

Efforts to minimize surgical trauma have driven advancements in minimally invasive surgery (MIS) [3, 4]. Among various MIS approaches, Natural Orifice Transluminal Endoscopic Surgery (NOTES) represents an innovative yet debated technique [3–6]. NOTES utilizes natural orifices (e.g., mouth, rectum, urethra, vagina) to perform endoscopic procedures without visible abdominal scars.11 Its feasibility was first demonstrated in 2003 with a transgastric appendectomy [7]. Since then, NOTES has been applied in cholecystectomy [8], gastrostomy [9], nephrectomy [10], oophorectomy [11], and ovarian cystectomy [12, 13]. Its advantages include reduced postoperative pain, minimal hemorrhage, and improved cosmesis [14, 15]. However, concerns regarding limited operative space, difficult suturing, and steep learning curves remain [16]. 

Transvaginal NOTES (vNOTES) mitigates some challenges associated with other NOTES approaches and is increasingly preferred in gynecology [17–19]. The vaginal route offers favorable anatomical characteristics, including good ductility, strong healing ability, ample operative space, and proximity to target organs [16, 20]. Since Zorron et al. first performed transvaginal endoscopic cholecystectomy [21], vNOTES has been applied to a range of benign gynecologic conditions [20, 22–25]. It has even been explored in the treatment of early-stage gynecologic malignancies [26]. A previous study on hysterectomy and bilateral adnexectomy in transgender men also demonstrated that vNOTES is a feasible and safe alternative to laparoscopic surgery [27]. 

Despite its potential, concerns remain regarding vNOTES’ impact on sexual function, pregnancy, vaginal delivery, adjacent organ damage, and incisional complications [14, 15]. Several retrospective studies have assessed vNOTES’ safety and effectiveness, though the lack of RCTs introduces potential bias [14, 20, 28–31]. Existing RCTs, such as the HALON and NOTABLE studies [23, 24, 32, 33], focus on hysterectomy and adnexectomy, leaving gaps in evidence for tubal ectopic pregnancy.

Our institution has extensive experience with vNOTES, performing 500–600 cases annually, alongside 1000–2000 TU-LESS procedures. Retrospective studies suggest vNOTES has lower perioperative complication rates but slightly higher conversion rates than TU-LESS [25, 34]. Given the similarities and controversies between these approaches, we designed the vNOTESTEP study to systematically evaluate vNOTES’ feasibility, safety, and long-term outcomes in treating tubal ectopic pregnancy.

## Methods

### Study design

The vNOTESTEP study is a single-blind RCT designed to evaluate the perioperative and long-term postoperative outcomes of vNOTES compared withTU-LESS in patients with tubal ectopic pregnancy. Considering ethical concerns and the low likelihood that patients would be unaware of their incision site, this study will adopt a single-blind design, with blinding applied only to the outcome assessors (OAs) and not to the patients. After obtaining informed consent from each participant, preoperative assessments will be conducted. Following randomization, salpingectomy will be performed via either vNOTES or TU-LESS approach. The primary outcomes include perioperative outcomes like surgical conversion, change of postoperative sexual function, pregnancy, and vaginal delivery. The study will be conducted at CWCCH, a university teaching hospital and regional training center for vNOTES, spanning from 2024 to 2030, with two years allocated for recruitment and three to five years for follow-up.

CWCCH has a highly experienced team of approximately 20 gynecologic endoscopic specialists, who collectively perform 500–600 vNOTES and 1000–2000 TU-LESS procedures annually. Randomization and grouping will be performed by a cohort manager (CM) using a computer-generated system after stratification. To maintain blinding, identifiable patient information will be removed. Outcome assessors (OAs) will be blinded to patient allocation, with OA1 responsible for evaluating incisional wounds and OA2 assessing non-incisional wound-related perioperative and postoperative outcomes. Both the umbilical and vaginal incisional sites will be dressed uniformly to achieve blinding during hospitalization. Patients will not be informed of the type of surgery they undergo either preoperatively or postoperatively to maintain blinding as much as possible. In cases of severe incisional complications, blinding may be lifted for appropriate medical intervention. Information exchange between CM, OAs, and cohort coordinators (CCs) will be restricted until the study’s completion.

### Study population

#### Sample size calculation

Sample size estimation was based on the NOTABLE cohort study [24]. Previous studies report a conversion rate for vNOTES in tubal ectopic pregnancies between 1.83% and 3.07%, suggesting a surgical success rate of approximately 95%.^34,35^A one-sided non-inferiority test was used to determine the sample size, assuming that vNOTES would remain a preferred option despite a potential 15% lower success rate compared to TU-LESS, which results in visible scarring. To achieve 85% power for non-inferiority with a 95% expected success rate in both groups, a total of 54 cases were required. Accounting for an anticipated 20% dropout rate, the final sample size was set at 72 participants, with 36 per group.

### Patient selection

#### Inclusion criteria

1. Diagnosis of tubal ectopic pregnancy.
2. Sign informed consent.
3. Age ≥ 18 years.
4. Indications for laparoscopy.

#### Exclusion criteria

1. No history of sexual activity.
2. Suspected pelvic infection.
3. Vaginal stenosis or vaginitis.
4. Severe pelvic adhesions.
5. History of hernia or rectal surgery.

### Study interventions

Both vNOTES and TU-LESS procedures will follow standardized surgical techniques described in previous literature [34, 35]. All procedures will be performed by a team of experienced gynecologic endoscopic specialists. Among them, 10 surgeons have over 20 years of experience, each performing more than 100 TU-LESS and 50 vNOTES procedures annually, while the remaining 10 surgeons have over 10 years of experience, performing 50–100 TU-LESS and 25 vNOTES cases per year. All surgeons have completed their learning curves.

### Recruitment procedures and follow-up plan

#### Recruitment

**Fig. 1:**
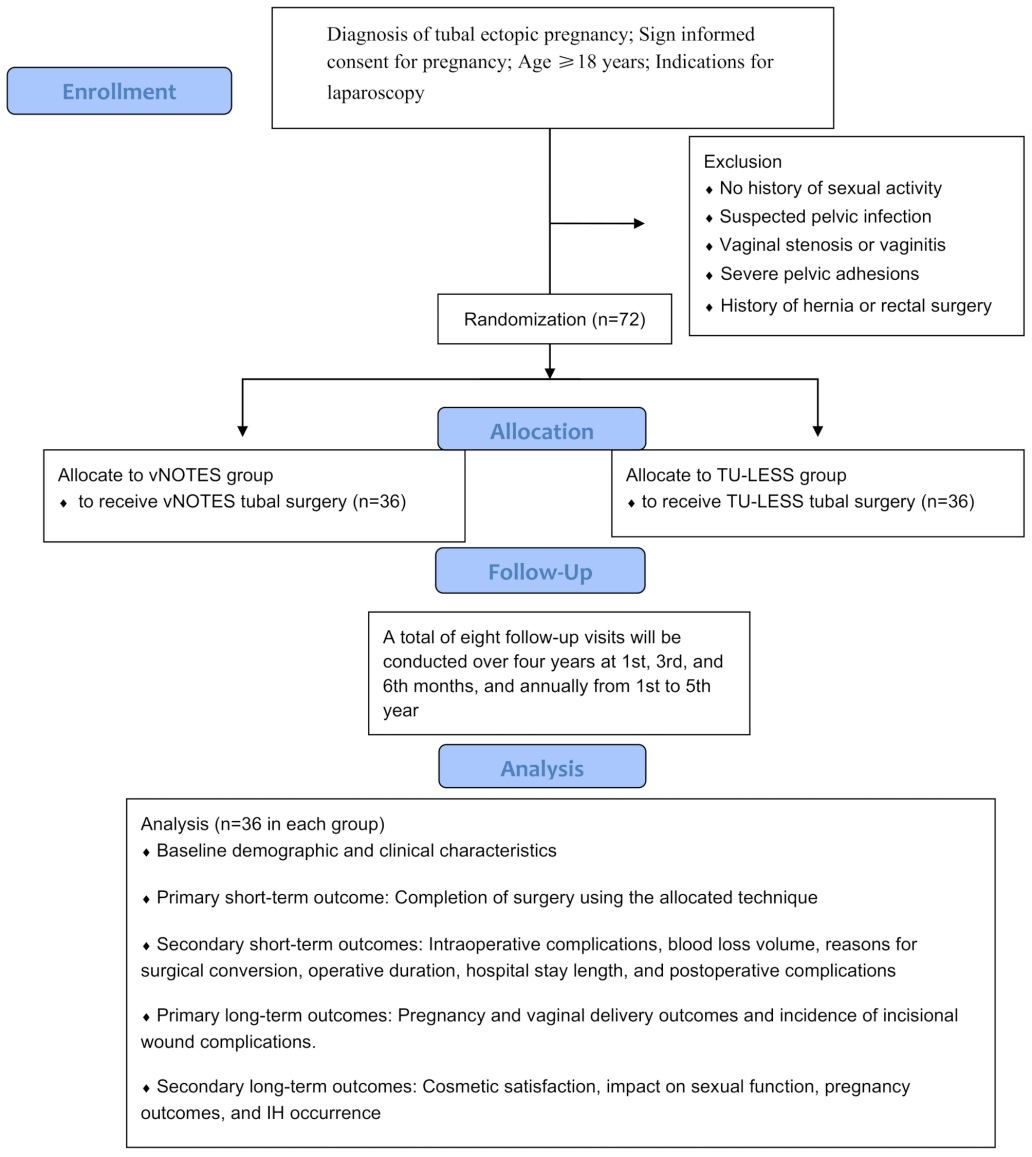
Schematic diagram presenting the flowchart of the vNOTESTEP study. Abbreviations: vNOTESTEP, Transvaginal Natural Orifice Transluminal Endoscopic Surgery for Tubal Ectopic Pregnancy study; vNOTES, Transvaginal Natural Orifice Transluminal Endoscopic Surgery; TU-LESS, transumbilical laparoendoscopic single site; IH, incisional hernia

**Fig. 2:**
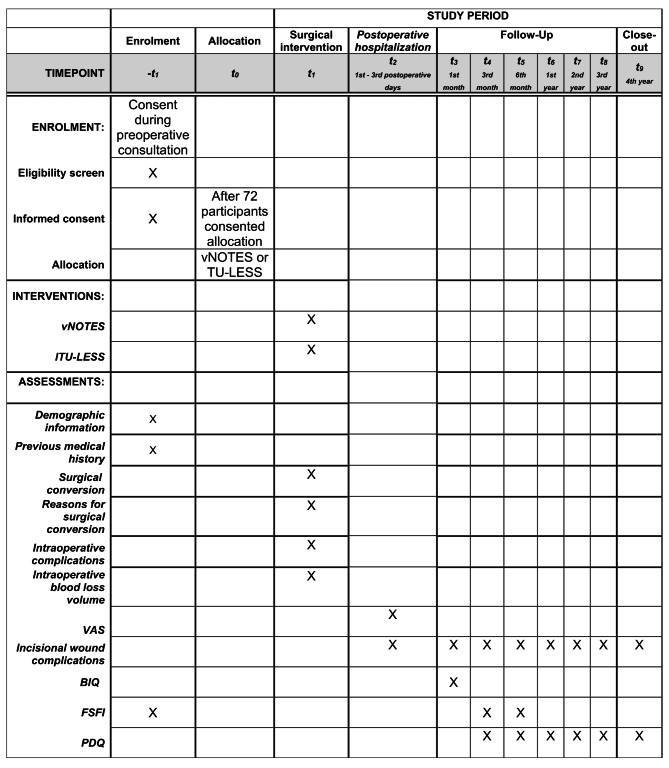
Schedule of enrolment, interventions, and assessments of the vNOTESTEP study. Abbreviations: vNOTESTEP, Transvaginal Natural Orifice Transluminal Endoscopic Surgery for Tubal Ectopic Pregnancy study; vNOTES, Transvaginal Natural Orifice Transluminal Endoscopic Surgery; TU-LESS, transumbilical laparoendoscopic single site; VAS, Visual Analog Scale; BIQ, Body Image Questionnaire; PDQ, Pregnancy and Delivery Questionnaire; IH, incisional hernia

The enrolment flowchart and follow-up plan for the vNOTESTEP study are presented in Figs. 1 and 2. Three trained cohort coordinators (CCs) will provide standardized vNOTES and TULESS-related information to potential participants, outlining study requirements, including multiple follow-up visits and questionnaire completion. Patients will be enrolled upon signing informed consent, after which baseline data will be collected from the Hospital Information System (HIS).

#### Perioperative management

A preoperative assessment will determine the appropriate surgical approach. Intraoperative and short-term postoperative data will be recorded in the HIS. Preoperative Female Sexual Function Index (FSFI) and postoperative Visual Analog Scale (VAS) scores will also be collected. WeChat groups will be established for patient education, management, and follow-up, with patients invited to join before hospital discharge.

#### Postoperative follow-up

A total of eight follow-up visits will be conducted over four years at 1, 3, and 6 months, and annually from years 1 to 4. Standardized questionnaires and clinical assessments will be administered as presented in Fig. 2. Completed questionnaires will be automatically uploaded to the study database. Ultrasound and gynecologic physical examinations will be performed when necessary.

### Quality control and cohort maintenance

CCs will assess questionnaire responses for completeness and accuracy. If deficiencies are identified, participants will be requested to re-complete the forms. To minimize loss to follow-up, incentives such as free clinic visits, partial reimbursement of examination fees, and expedited appointments will be provided. CCs will regularly disseminate healthcare tips, educational materials, and reminders via WeChat. The cohort manager (CM) will be available for direct online medical consultations. Participants lost to follow-up will be contacted via phone or WeChat to encourage continued participation.

### Items of follow-up visits

Follow-up assessments in the vNOTESTEP study will include standardized scales, questionnaires, and clinical investigations. The Chinese versions of all questionnaires used in this study were professionally translated and validated via independent back-translation. The following measures will be used to evaluate postoperative outcomes of vNOTES and TU-LESS:

- **Body Image Questionnaire (BIQ)**: An eight-item scale assessing postoperative body image and cosmetic perception. Items 1–5 evaluate patients’ self-perception and satisfaction with their appearance, while items 6–8 assess satisfaction with the surgical scar [36, 37]. BIQ will be administered at the 1-month follow-up visit.
- **Visual Analog Scale (VAS)**: A validated tool for pain assessment, consisting of a 10 cm-long scale ranging from ‘no pain’ to ‘extreme pain.’ Patients will indicate pain intensity on postoperative days 1, 2, and 3. Pain scores will be classified as: <1 cm (painless), 1–3 cm (mild), 4–6 cm (moderate), and 7–10 cm (severe). CCs will record and upload VAS scores to the study database.
- **Female Sexual Function Index (FSFI)**: A validated 19-item scale assessing six domains of sexual function, including libido, arousal, lubrication, orgasm, satisfaction, and pain [38]. A total score < 26.5 indicates impaired sexual function [39]. The FSFI will be administered preoperatively and postoperatively at 3 and 6 months. Since sexual activity is contraindicated for one month following vNOTES to allow vaginal incision healing, FSFI will not be assessed at earlier time points [40].
- **Pregnancy and delivery outcomes**: A self-designed **Pregnancy and Delivery Questionnaire (PDQ)** collecting postoperative gestational history (see Appendix 1). The PDQ includes seven questions regarding pregnancy status, conception method, delivery mode, parity, vaginal laceration, and incisional hernia (IH). This will be administered at all follow-up visits except the 1st month. Patients will be encouraged to undergo pregnancy monitoring and delivery at CWCCH to facilitate comprehensive data collection via HIS.
- **Incisional complications**: At each follow-up visit, patients will undergo gynecologic physical examinations to assess for IH. Patients with suspected IH (i.e., those experiencing incisional pain or a bulge) will be referred for imaging investigations, including ultrasound, computed tomography (CT), or magnetic resonance imaging (MRI). Diagnosed IH cases will be treated appropriately, and CCs will document relevant findings in the study database.

### Data collection and management

Gynecologists from inpatient and outpatient departments will document preoperative and surgical assessments in the HIS. Face-to-face interviews or phone calls will be conducted to supplement data collection. A gynecologic laparoendoscopic specialist will serve as the CM, overseeing cohort management and CCs’ responsibilities. CCs will obtain informed consent, conduct follow-up visits, and facilitate data entry into the study database.

To minimize bias, assessors will remain blinded to patients’ surgical allocation. The CM will ensure that surgical approach details are concealed before CCs collect perioperative and follow-up data. Randomization will also be applied to CCs’ work assignments. A secure, self-built database will store all study data.

### Baseline information

Baseline demographic and clinical characteristics of enrolled participants will be recorded in the HIS, including age, education level, occupation, household income, lifestyle factors (smoking, alcohol consumption), comorbidities, medication history, reproductive and surgical history, height, weight, and body mass index (BMI).

### Perioperative assessment and short-term postoperative outcomes

#### Primary short-term outcome

Completion of surgery using the allocated technique is Primary short-term outcome that we will focus on. We will also conduct a detailed analysis of cases requiring surgical conversion, including the reasons for conversion, the type of surgical techniques converted into, as well as the specific complications and their severity.

#### Secondary short-term outcomes

Intraoperative complications (e.g., unintended organ injury), blood loss volume, reasons for surgical conversion, operative duration, hospital stay length, and postoperative complications (e.g., bleeding, wound infection, pain, urinary retention, febrile morbidity). Perioperative complications will be classified according to the Clavien–Dindo system [41]. 

### Long-term postoperative outcomes

#### Primary long-term outcomes

Pregnancy and vaginal delivery outcomes and incidence of incisional wound complications.

#### Secondary long-term outcomes

Cosmetic satisfaction, impact on sexual function, pregnancy outcomes, and IH occurrence. These outcomes will be evaluated according to the follow-up schedule outlined in Figs. 1 and 2.

### Economic evaluation

A cost-effectiveness analysis will compare hospitalization, surgery, and consumable costs between vNOTES and TU-LESS. Findings will support guideline development and inform patient decision-making.

### Statistical analysis

Statistical analyses will be conducted using SPSS (Version 25.0) and R. Continuous variables will be analyzed using t-tests or Mann–Whitney tests, with results presented as means ± standard deviations for normally distributed data and medians with interquartile ranges for non-normally distributed data. Categorical variables will be analyzed using chi-square or Fisher’s exact tests. To mitigate bias, statistical techniques such as propensity score matching (PSM), stratification, multivariate analysis, Cox regression, and generalized estimating equations (GEE) will be employed as necessary. A binary logistic regression analysis will also be conducted to assess the risk factors for surgical conversion, incorporating variables that demonstrate a significant statistical difference in the univariate comparisons. Statistical significance will be set at *p* < 0.05.

## Discussion

Tubal ectopic pregnancy poses a significant risk to maternal health and remains a leading cause of early pregnancy morbidity. Minimally invasive laparoscopic techniques are increasingly preferred for its management [2, 42]. Given its multiple advantages in treating benign gynecologic conditions, vNOTES has gained popularity among gynecologic endoscopic specialists [18]. Several retrospective studies indicate that vNOTES for tubal ectopic pregnancy and other benign gynecologic conditions, including emergency cases, is associated with reduced perioperative complications, superior cosmetic outcomes, decreased postoperative pain, and a faster recovery compared to conventional laparoscopic techniques [22, 34, 35, 43–45]. However, the lack of RCT design, comprehensive long-term follow-up data, and concerns regarding the financial burden of vNOTES may limit its widespread adoption [14, 15, 29]. 

The present study aims to compare vNOTES and TU-LESS due to their shared minimally invasive nature and similar clinical indications. TU-LESS has been increasingly adopted as an alternative to CL and MPL in benign gynecologic disease with surgical indications, making it a relevant comparator in the context of minimally invasive gynecologic surgery. At our institution, vNOTES and TU-LESS are preferred over CL and MPL due to their aesthetic and other advantages. Additionally, the economic costs of these surgical techniques do not differ significantly. Most patients opt for either vNOTES or TU-LESS, with the number of patients choosing vNOTES being approximately twice that of TU-LESS. A similar trend has also been observed in other countries with distinct cultural backgrounds compared to China [17]. Due to the greater invasiveness of CL, it is primarily reserved for intraoperative conversion in cases where severe complications prevent the completion of vNOTES or TU-LESS as initially planned. Moreover, previous studies have extensively compared MPL with vNOTES or TU-LESS [43, 45, 46]. For these reasons, our study, as well as other RCTs in the field of minimally invasive gynecologic surgery, aims to address a gap in the literature by specifically focusing on the comparison between vNOTES and TU-LESS rather than other surgical methods [23, 24, 32, 33, 47]. 

Patient perceptions of vNOTES have been extensively explored, with primary concerns relating to the necessity of culdotomy and its potential effects on incisional complications, sexual function, pregnancy, and vaginal delivery [48–50]. A retrospective study of 76 patients with a median follow-up of 77 months found no significant impact of hybrid-NOTES on pregnancy outcomes or delivery mode [51]. However, this study primarily involved transvaginal specimen retrieval rather than full vNOTES procedures. A more recent study reported that most women undergoing vNOTES achieved normal pregnancy and vaginal delivery, but the small sample size (*n* = 9) limits its statistical power and generalizability [28]. A prospective cohort study comparing the perioperative outcomes of vNOTES and TU-LESS hysterectomy involving 192 patients also found that the vNOTES group demonstrated several advantages over the TULESS group, including shorter operative time, faster postoperative recovery, reduced hospital stay, greater minimally invasive benefits, and improved cosmetic outcomes. However, intraoperative blood loss was greater in the vNOTES group [52]. While some RCTs comparing vNOTES and TU-LESS have been initiated [23, 24, 33], their follow-up duration and scope remain limited. Additionally, RCTs assessing the impact of vNOTES on sexual function have largely focused on non-gynecologic applications in non-Chinese populations, with few employing a single-blind RCT design [53–55]. 

Another key aspect of the vNOTESTEP study is the incidence of postoperative incisional hernia (IH), a topic with limited existing research. Studies on TU-LESS indicate an IH incidence of 5–7%, rising to 1–30% in obese patients [56, 57]. However, these findings cannot be directly applied to vNOTES due to anatomical differences in surgical access points. Given its incision site, vNOTES may theoretically reduce IH rates in obese patients, but clinical evidence is needed to substantiate this hypothesis.

Low postoperative pain is one of the advantages of vNOTES. Up to 80% of patients undergoing conventional laparoscopic surgery report certain levels of pain that requires analgesia. The origins of post-laparoscopic pain are multifactorial, including inflammatory responses linked to surgical trauma and incision sites, as well as structural and biochemical changes in the peritoneum and diaphragm induced by pneumoperitoneum. The latter mechanism results from irritation, mechanical stretching, and foreign body stimulation, which can trigger phrenic nerve dysfunction and lead to subsequent shoulder-tip pain [58]. Since the surgical incision in vNOTES is primarily located at the posterior vaginal fornix, an area with relatively sparse nerve distribution, vNOTES theoretically outperformed TU-LESS in reducing postoperative pain. Consistently, numerous retrospective studies have demonstrated significantly lower postoperative VAS pain scores in vNOTES compared to TU-LESS or MPL [17, 31, 43–45, 59, 60]. However, high-quality evidence from RCTs remains limited.

The steep learning curve of vNOTES has also been cited as a barrier to its adoption.9 However, it was reported that with adequate training and experience, the surgical setup time can be reduced in most cases. Additionally, vNOTES may offer advantages over TU-LESS in obese patients, as evidenced by a case report describing its successful application in cholecystectomy for a morbidly obese patient [61]. 

Technological advancements have significantly enhanced the feasibility and efficiency of vNOTES. Innovations such as prone-position vNOTES for posterior uterine wall myomectomy [62], robot assisted vNOTES [63, 64], as well as the growing compatibility between vNOTES and TU-LESS instrumentation, continue to refine the technique. As TU-LESS technology progresses, parallel advancements in vNOTES are expected, potentially overcoming existing technical limitations.

Despite these advantages, there remain concerns regarding the impact of vNOTES on reproductive outcomes. Given the transvaginal access route, potential alterations in vaginal elasticity, microbial environment, and long-term pelvic floor function should be considered. Additionally, vNOTES could introduce theoretical risks of adhesion formation due to peritoneal exposure, though existing studies have yet to establish a definitive association. Further research is necessary to delineate these potential risks and to confirm whether vNOTES remains a preferable option for patients desiring future fertility.

Economic considerations are another critical factor influencing the adoption of vNOTES. While vNOTES provides superior cosmetic results and potentially lower postoperative pain, its cost-effectiveness compared to TU-LESS remains a point of debate. The high initial cost of specialized instrumentation and training requirements may deter widespread implementation, particularly in resource-limited settings. Future cost-effectiveness analyses incorporating long-term patient outcomes and healthcare expenditures will be essential for assessing the broader viability of vNOTES in clinical practice.

Furthermore, public perception and patient acceptability play a pivotal role in determining the clinical adoption of novel surgical techniques. Cultural factors and varying levels of awareness about vNOTES may impact on patients’ willingness to undergo this procedure. Studies have indicated that patient education and preoperative counseling significantly influence decision-making, and further efforts should be directed toward enhancing patient understanding of vNOTES’ benefits and potential risks.

Some previous RCTs comparing vNOTES and TU-LESS in treating gynecologic diseases either did not implement blinding or used non-therapeutic incisions for blinding. However, the later approach is controversial, as non-therapeutic incisions may harm patient welfare and introduce ethical concerns [24, 47]. Additionally, such extraneous incisions could create a confounding effect on aesthetic and postoperative pain assessments, potentially influencing the evaluation of postoperative outcomes for vNOTES and TU-LESS. Although we did not plan to inform patients of the surgical procedure which they will undergo either preoperatively or postoperatively to achieve blinding as much as possible, we acknowledge that due to the visibility of the umbilical incision, which can be easily detected by the patient, the effectiveness of blinding may be compromised. After careful consideration, we believe that the current single-blind design in our study strikes an acceptable balance between the effectiveness of blinding and patient welfare.

A notable limitation of the study is its single-center design and focus on a regional Chinese population, which may affect generalizability. Moreover, restricting the study to TU-LESS and vNOTES, without including other surgical techniques, may limit the applicability of the results. We also acknowledge that the sample size may be insufficient to detect rare complications, therefore a prospective study on the vNOTES and TU-LESS’ surgical outcomes in treating ectopic tubal pregnancy with larger sample size is warranted. Nonetheless, this two-arm, prospective RCT with an extensive long-term follow-up plan offers a valuable opportunity to refine vNOTES protocols, inform clinical guidelines, and improve patient acceptance of this minimally invasive approach.

In conclusion, the vNOTESTEP study is designed to generate high-quality evidence from an RCT on the perioperative and long-term outcomes of vNOTES in treating tubal ectopic pregnancy, with a particular focus on sexual function, pregnancy outcomes, vaginal delivery, and the incidence of incisional hernia.

## Electronic supplementary material

Below is the link to the electronic supplementary material.

Supplementary Material 1

## Data Availability

No datasets were generated or analysed during the current study.

## Abbreviations

BIQ: Body Image Questionnaire
BMI: Body mass index
CC: Cohort Coordinator
CM: Cohort Manager
CT: Computer Tomography
CWCCH: Chengdu Women’s and Children’s Central Hospital
FSFI: Female Sexual Function Index
GEE: Generalized estimating equations
HALON: Hysterectomy by transabdominal laparoscopy or natural orifice transluminal endoscopic surgery
HIS: Hospital Information System
IH: Incisional Hernia
MIS: Minimally Invasive Surgery
MPL: Multiport Laparoscopic Surgery
MRI: Magnetic Resonance Imaging
NOTABLE: Transvaginal natural orifice transluminal endoscopic surgery (vNOTES) adnexectomy for benign pathology compared with laparoscopic excision
OA: Outcome Assessors
PDQ: Pregnancy and Delivery Questionnaire
PROMs: Patient Reported Outcome Measures
RCT: Randomized Control Rial
TU-LESS: Transumbilical Laparoscopic Single Site Surgery
VAS: Visual Analog Scale
vNOTES: Transvaginal Natural Orifice Transluminal Endoscopic Surgery
vNOTESTEP: Transvaginal Natural Orifice Transluminal Endoscopic Surgery in Treating Ectopic Pregnancy cohort study
