# Supplementary material for: Transvaginal natural orifice transluminal endoscopic surgery for tubal ectopic pregnancy(vNOTESTEP): a protocol for a randomized controlled trial
Source: BMC Pregnancy Childbirth. 2025 Apr 23;25:477. doi: 10.1186/s12884-025-07595-z (PMC12020209; doi:10.1186/s12884-025-07595-z)
Supplement: Supplementary file 1 — Supplementary Material 1 [file 12884_2025_7595_MOESM1_ESM.doc]

Pregnancy and Delivery Questionnaire

1. Did you get pregnant after the surgeries?
2. If you did get pregnant, do you get pregnant spontaneously or by IVF?
3. Is that a monoparous or multiparous pregnancy?
4. If you had delivered, what is your delivery manner? Vaginal delivery or [cesarean section](../../../../C:/Users/FengDan/AppData/Local/youdao/dict/Application/8.9.9.0/resultui/html/index.html#/javascript:;)?
5. If you had delivered, have you ever had vaginal incision hernia or other pathological situation related to vaginal incision during pregnancy?
6. Have your attempted a vaginal delivery or chosen [cesarean section](../../../../C:/Users/FengDan/AppData/Local/youdao/dict/Application/8.9.9.0/resultui/html/index.html#/javascript:;) [voluntarily](../../../../C:/Users/FengDan/AppData/Local/youdao/dict/Application/8.9.9.0/resultui/html/index.html#/javascript:;)?
7. If you had vaginally delivery, have you experienced vaginal laceration during vaginal labor? If had, what’s the details?
